# Tetraploidy and tumor development

**DOI:** 10.18632/oncotarget.2790

**Published:** 2014-11-19

**Authors:** Sanghee Lim, Neil J. Ganem

**Affiliations:** Shamim and Ashraf Dahod Breast Cancer Research Laboratories, Departments of Pharmacology & Experimental Therapeutics and Medicine, Division of Hematology and Oncology, Boston University School of Medicine, Boston, MA, USA

Tetraploid cells, which contain a doubled chromosomal content, are known to facilitate tumorigenesis [1]. Two specific characteristics of tetraploid cells play major roles in promoting neoplastic transformation. First, proliferating tetraploid cells are genomically unstable and accumulate both numerical and structural chromosomal abnormalities. Such chromosomal instability arises mainly due to the presence of supernumerary centrosomes that disrupt normal mitotic spindle assembly and chromosome segregation [2]. Second, extra copies of chromosomes in tetraploid cells act as a compensatory buffer against spontaneously arising deleterious mutations. This enables nascent tumor cells to continue proliferating in the presence of normally lethal genomic alterations [3]. Together, these two characteristics allow tetraploid cells to continuously sample multiple genetic permutations, ultimately giving rise to rare cells that have acquired growth advantages. Indeed, mounting evidence suggests that tetraploidy may have a significant and previously unappreciated role in the development of solid tumors. Computational analysis of sequencing data from ~4000 human cancers has indicated that approximately 40% of all human tumors have undergone a tetraploidization event at some point during their progression [4]. This is true even of mature tumors that ultimately stabilize with a near-diploid complement of chromosomes. As such, while near-tetraploid tumors are less commonly seen in the clinical setting than their near-diploid or triploid counterparts, these observations are not necessarily indicative of the extent to which tetraploidy drives tumor development in patients.

However, several critical questions regarding tetraploid-driven tumor development remain unanswered. Though many tumors show evidence of having passed through a tetraploid stage at some point during the oncogenic process, it remains unclear how often such a stage acts as an intermediate preceding tumorigenesis, rather than arising as a secondary consequence of malignant transformation.

It also remains unresolved how often, and by what mechanisms, tumor-initiating tetraploid cells arise in pre-neoplastic tissues. Tetraploidy generally arises as a result of three different pathways: endoreduplication, in which the genome is re-replicated without an intervening mitosis; cell fusion, which is often instigated by viral infections; and cytokinesis/mitotic failure. Of the three pathways, cytokinesis or mitotic dysfunction is thought to be the most common way by which human cells become tetraploid. This is due in large part to the fact that a significant number of proteins are essential to complete the complicated process of cell division. Furthermore, oncogene-induced replication stress or telomere crisis, two common initiating events in tumor development, are also known to lead to cytokinesis failure by promoting the formation of chromosome bridges that occlude the ingressing cytokinetic furrow.

**Figure:**
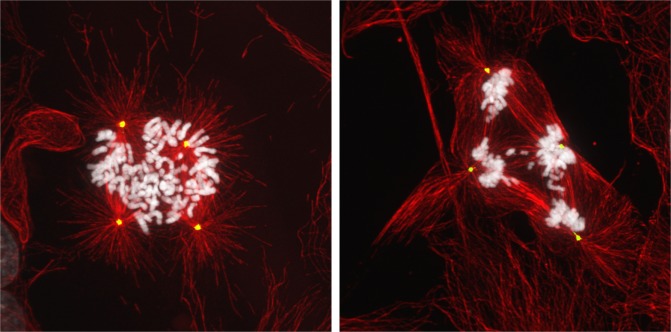
Tetraploid cells are chromosomally unstable

Finally, it remains unclear what role tetraploidy may play in tumor progression. As stated above, a direct consequence of tetraploidy is the acquisition of excess centrosomes, which imparts cells with chromosome instability. Chromosomal instability is known to promote tumor growth, as well as relapse following chemotherapeutic treatment [5]. Furthermore, the presence of extra centrosomes confers invasive-like behavior to cells [6]. These features provide some perspectives on how tetraploidy might promote neoplastic disease progression vis-à-vis direct phenotypic alterations closer to that of invasive carcinomas.

In light of the oncogenic potential of tetraploidy, it is unsurprising, then, to find that tumor suppression mechanisms have evolved to both sense the presence of tetraploid cells as well as limit their proliferation. Recent work has revealed the mechanistic basis for this growth arrest, as tetraploid cells have been shown to activate the Hippo tumor suppressor pathway, both *in vitro* and *in vivo* [7]. The Hippo pathway is an evolutionarily conserved pathway which controls cell proliferation by negatively regulating the transcriptional co-factors YAP and TAZ through phosphorylation by LATS1/2 kinases. New work reveals that tetraploid cells generated by cytokinesis failure activate LATS2, which both inactivates YAP/TAZ and promotes p53 stabilization by inhibiting MDM2 – thus enforcing tetraploidy-induced arrest. Inactivation of the Hippo pathway, either by depletion of LATS2 or by expression of a constitutively active version of YAP, is sufficient to restore proliferative capacity to tetraploid cells. The underlying mechanism for LATS2 activation in tetraploid cells can be largely attributed to diminished levels of active RhoA and reductions in actin contractility, both of which are well-described triggers of the Hippo pathway. This reduction in levels of active RhoA arises, at least in part, from the presence of extra centrosomes in tetraploid cells [7].

The clinical significance of these findings is not trivial. Hippo pathway inactivation is a key characteristic of many human cancers, and is significantly more common in high-ploidy tumors [7]. It is tempting, then, to speculate that inactivation or bypass of the Hippo pathway may be a prerequisite for the development of high-ploidy tumors. However, our understanding of the mechanisms by which cancer cells functionally inactivate the Hippo pathway remain vastly incomplete, as mutations in key components of the pathway are exceedingly rare. As such, identifying new regulators of Hippo signaling, and deciphering whether they are commonly dysregulated in human cancers, remains paramount. Characterization of mechanisms that underlie Hippo pathway activation – and inactivation – may uncover new potential avenues for rationally designed antineoplastic therapies which selectively target abnormal high-ploidy cancer cells while sparing normal healthy diploid cells.
